# Reptation-Induced Coalescence of Tunnels and Cavities in *Escherichia Coli* XylE Transporter Conformers Accounts for Facilitated Diffusion

**DOI:** 10.1007/s00232-014-9711-7

**Published:** 2014-08-28

**Authors:** Philip Cunningham, Richard J. Naftalin

**Affiliations:** 1 Department of Bioinformatics, School of Medicine, King’s College London, Waterloo Campus, Franklin–Wilkins Building, London, SE1 9NH UK; 2 Department of Physiology, School of Medicine, King’s College London, Waterloo Campus, Franklin–Wilkins Building, London, SE1 9NH UK; 3 BHF Centre of Research Excellence, School of Medicine, King’s College London, Waterloo Campus, Franklin–Wilkins Building, London, SE1 9NH UK

**Keywords:** Xylose, XylE structure, GLUT1, Facilitated diffusion, Docking

## Abstract

Structural changes and xylose docking to eight conformers of *Escherichia Coli* XylE, a xylose transporter similar to mammalian passive glucose transporters GLUTs, have been examined. Xylose docks to inward and outward facing conformers at a high affinity central site (*K*_*i*_ 4–20 µM), previously identified by crystallography and additionally consistently docks to lower affinity sites in the external and internal vestibules (*K*_*i*_ 12–50 µM). All these sites lie within intramolecular tunnels and cavities. Several local regions in the central transmembrane zone have large positional divergences of both skeleton carbon Cα positions and side chains. One such in TM 10 is the destabilizing sequence G388-P389-V390-C391 with an average RMSD (4.5 ± 0.4 Å). Interchange between conformer poses results in coalescence of tunnels with adjacent cavities, thereby producing a transitory channel spanning the entire transporter. A fully open channel exists in one inward-facing apo-conformer, (PDB 4ja4c) as demonstrated by several different tunnel-finding algorithms. The conformer interchanges produce a gated network within a branched central channel that permits staged ligand diffusion across the transporter during the open gate periods. Simulation of this model demonstrates that small-scale conformational changes required for sequentially opening gate with frequencies in the ns-μs time domain accommodate diffusive ligand flow between adjacent sites with association–dissociation rates in the μs-ms domain without imposing delays. This current model helps to unify the apparently opposing concepts of alternate access and multisite models of ligand transport.

**Electronic supplementary material:**

The online version of this article (doi:10.1007/s00232-014-9711-7) contains supplementary material, which is available to authorized users.

## Introduction

The nature of facilitated ligand transport remains unresolved. Conventionally sugar transport via the major facilitator superfamily (MFS), e.g. GLUTs, is viewed as a process where ligands reversibly bind at a centrally located site and are transported between the inside (cytosolic) and external (periplasmic) face of the transporter by positional changes of the transmembrane helices (Carruthers et al. 2009; Madej et al. 2013) that lead to alternate opening and closure of gates to ligand transit. This process relocates the bound ligand to the alternate side, whilst maintaining transporter occlusion and thereby preventing back leakage. Crystallography of transporter proteins shows inward and outward facing conformations of various transporter proteins with the ligand centrally bound at sites that are occluded or open to the internal or external solution (Smirnova et al. 2011). It has been speculated that the major conformational changes that result in inversion around the central binding site are correlated with dynamic mechanisms of ligand transport, thus lending support to the alternating access transport model (Gadsby 2009).

However, the precise role of the conformational changes in the mechanics of the transport process remains obscure. Molecular dynamics extending to the millisecond time domain has recently shown that conformational changes required to simulate net and exchange water movements between external solution and hydration sites within cavities of small proteins such as bovine pancreatic trypsin inhibitor and myoglobin occurs in regions with higher than average flexibility (Persson and Halle 2008; Shaw et al. 2010; Persson and Halle 2013). Although it is suggested that prolonged molecular dynamic simulations of large proteins, such as membrane transporters will be possible within the foreseeable future, no evidence exists currently to show as has been envisaged by several groups (Shimamura et al. 2010; Kaback et al. 2011; Weyand et al. 2011; Radestock and Forrest 2011; Solcan et al. 2012) that conformational inversion at a single central site is the main or even a significant mode of facilitated transport.

A number of phenomena are inconsistent with the central site being the only determinant of ligand selectivity and transport. Two aberrant GLUT1 mutants causing glucose transporter deficiency syndrome, GLUT1DS in human infants generate a temperature sensitive defect in glucose transport that is observed in transfected *xenopus* oocytes at 37 °C, but not in the patients’ erythrocytes at 4 °C (Wang et al. 2008). The mutant transporter results in larger functional changes in glucose affinity on the exporting inside face of the transporter than for import, on the outside face and thereby alters the normal asymmetry ratio from ten-fold lower affinity inside to lower affinity outside than inside (Cunningham et al. 2006; Cunningham and Naftalin 2013). These aberrant GLUT1DS mutations are positioned in the linker region between TMs 7 and 8 in the external vestibular surface of the transporter, whilst exerting their functional effects mainly on the exporting affinity site, which the alternating access model locates on the inside of the transporter. This is an inconsistency because this model requires ligand selectivity sites to be centrally located. The central location is required so that the transported ligand can access the alternate side of the transporter with minimal rearrangements of the transporter. If the main binding site is at either end of the transport, then a much larger conformational change would be required for the transported ligand to gain access to the alternate side. It remains plausible that a mutant site positioned in an external linker region could prevent ligand access to the central binding and inverting site; so might be compatible with the alternating access model. However this would not explain the major functional inconsistency that the externally located mutation actually has a larger kinetic effect at the internally located export site.

Furthermore, the finding that glucose transport is asymmetric does not accord with a single cycle transport network, as required by the thermodynamic constraints of the single site alternating access model (Naftalin 2008a, 2010).

Additionally, as trivalent arsenicals are transported independently of glucose in GLUTs (Jiang et al. 2010) and water also permeates via parallel pathways to the primary ligand transport pathway in several other classes of transporter including GLUTs (Li et al. 2013), this is indicative of parallel or braided transport pathways via MFS transporters.

Moreover, where solute transport has been observed with patch clamp techniques, as with the dopamine transporter, it is evident that the normal slow ion and ligand transport can reversibly change to a higher ionic conductance and a much higher dopamine transport rate, suggesting that the transporter can transform transiently, in the millisecond time domain, into a channel (Kahlig et al. 2005). A recent molecular dynamic simulation of osmotic water transport across transporters shows similar episodic increases in permeability of the water conducting state (Li et al. 2013).

Our recent docking studies of d-glucose onto a 3D GLUT1 structure templated to the outward-facing conformer of XylE (PDB 4GBY) (Cunningham and Naftalin 2013) with the docking program Autodock Vina (Trott and Olson 2009) confirmed a previous study showing that in addition to the high affinity central docking site in the central core of the transporter, several additional lower affinity glucose binding sites are present in the external and internal vestibules (Cunningham et al. 2006). These lower affinity sites are less likely to be detected by crystallography (Davis et al. 2003). The presence of additional binding sites within the internal and external vestibules supports an alternative model for ligand transport, whereby ligands diffuse between multiple adjacent sites within a branched network across the transporter (Naftalin and De Felice 2012; Carruthers et al. 2009; Naftalin 2010) instead of a model where the ligand is exclusively bound centrally and requires a conformational change at the binding site before transport occurs. A recent molecular dynamics model suggests that the rocker-switch model requiring rigid transmembrane helices to propagate the conformational changes over the entire long axis of the transporter may not reflect the actual events occurring and an alternative “airlock” model has been suggested in which sequential gating occurs at either end of the transport channel (Stelzl et al. 2014).

Nevertheless, a key problem with reconciling this multisite model to known GLUT and other MFS structures e.g. *Escherichia Coli* LacY, is the existence of a narrow central region in these proteins, that prevents hydrophilic ligands like *N*-ethylmaleimide penetrating from the external solution and reacting with distal cysteine residues positioned, either naturally or by targeted mutation studies, in transmembrane (TM) regions (Mueckler and Makepeace 2010; Kaback et al. 2011). It is evident from studies with covalent ligand binding and fluorescence probes that conformational changes within this narrow central zone impede ligand access and transport across the transporter (Smirnova et al. 2011).

Two recent papers have presented crystallographic data of the 3D structure of the major facilitator (MFS) xylose transporter *Escherichia Coli XylE*. The first showed three outward holo-conformers, partially occluded with bound xylose PDB (4GBY, 4GBZ and 4GC0) at resolutions of 2.8 Å (Sun et al. 2012). The more recent paper shows five other conformers, two partially occluded holo-conformers: PDB (4JA3a and 4JA3b) in inward-facing conformations and three fully open inward-facing apo-conformers: PDB (4JA4a, 4JA4b and 4JA4c) at resolutions of 4.2 and 3.8 Å (Quistgaard et al. 2013).

Xylose transport via *XylE* has similar properties and functional characteristics to glucose transport via GLUT1 in human erythrocytes and other tissues. The protein has sequence identities of around 30 %, and similarities of around 50 %, with GLUTs1-4. The functional similarities include *saturation kinetics* and *accelerated exchange.* The *K*_m_ for d-xylose determined by transport and binding 0.47 ± 0.05 mM is affected by widely distributed missense mutations within the transporter (Sun et al. 2012). Some differences include failure to transport d-glucose and proton gradient sensitivity of sugar transport, which is not found in GLUTs1-4.

These newly available transporter structures provide an opportunity to investigate how xylose docking varies between the outward and inward-facing *XylE* conformers and also to determine the consistency of docking between conformers and whether the presence of these sites within the various conformers accords with current views on facilitated sugar transport.

## Methods

d-xylose binding to the eight available conformers of *XylE* has been investigated using Autodock Vina, an open sourced molecular docking program. vina.scripps.edu. The program replaces the hydrogen atoms that are missing from PDB files prior to docking. No other changes are made to the coordinates of the XylE crystal structures. Energy minimisation was not applied to these crystal structures by using sub routines, such as GROMOS, in Swissprot Viewer. In this current study this procedure was considered both unnecessary and counterproductive, as we wished to study xylose docking without any alterations the conformer variants.

The relationship of xylose docking within the intramolecular tunnels penetrating to the core of the protein was studied with the newly available surface and intramolecular cavity/tunnel mapping software in Swissprot Deepview PDB viewer, version 4.10. Two other tunnel and cavity detection programs “fpocket”, an open source program (fpocket.sourceforge.net/) software tool and Mole 2 (Sehnal et al. 2013), employing the Voronoi tessellation method, are used for analysing the extent, and positions and intramolecular tunnel dimension of XylE, provide additional independent corroboration.

The *Escherichia Coli* XylE structures were taken from the Protein Data Base (www.ebi.ac.uk/pdbe) using the PDB codes 4GBY, 4GBZ, 4GC0, 4ja3 and 4ja4. These crystal structures were published as trimer and dimer crystals, respectively. Each conformer structure was separated into individual chains which we term 4GBY, 4GBZ, 4GC0, 3a, 3b, 4a, 4b, and 4c respectively. The docking ligand molecules were prepared using Autodock Tools Version 1.5.4 Version 3 (Trott and Olson 2009). It could be also argued that some of the conformers within the multimeric crystals may be artifactual and indeed all of the conformers may differ from their conformations within lipid bilayers. However, to resolve this question would either require observing the crystals within the lipid bilayers; currently an unavailable option, or reassembling the crystal in silico within a virtual bilayer. Whilst perfectly feasible and clearly of some validity this would introduce another synthetic layer which might also distort the crystal structure and be considered inauthentic.

There are several unstructured regions in the lower resolution conformer structures 3a, 3b, 4a, 4b and 4c, the chains were ‘restructured’ using the UniProt sequence P0AGF4 (XylE_ECOLI) in SwissModel Automatic Modelling Mode at http://swissmodel.expasy.org. These newer structures were superimposable on the originals and did not alter any of the docking positions or assigned affinities of the docked ligands. There unstructured regions in the lower resolution conformers 3a, 3b, 4a,4b and 4c mainly occur in the exofacial linker regions and in some cases are close to docking sites in the external vestibule, so to some extent reduce the reliability of the estimated affinities of these external docking sites (see supplementary files 2). These unstructured linker regions may also affect the estimated volumes of both the inner and outer vestibules.

Unconstrained docking within a 15 or 20 Å sided cube, centred on the approximate middle of the targeted amino acid(s) was defined using Jmol’s (http://jmol.sourceforge.net/) bound box command. The xyz coordinates of each atom in XylE conformers were obtained from the PDB files. The relevant data sets are selected for position within the 3D XylE structure using Swissprot Deepview. The distances of the Cα atoms are all estimated with reference to carbon skeletal chain atoms in conformer 4Ja3a (3a) using the root mean square deviations RMSD obtained from the PDB coordinates. The affinities are assigned by the Autodock program. The absolute values of ligand affinity are based on the absolute temperature assignment at 300 °K. The estimates of relative affinities are more noteworthy than their absolute values.

RMSD of the Cα and side chains relative to those of PDB J3a were obtained from the relevant PDB files and estimated using self-generated algorithms to display these in Microsoft Excel worksheets. A comparison of the position deviations in all the side chains was undertaken by comparing the relative positions of all the most distal side chain atoms in all conformers relative to those in 3a; namely: ALA CB; ARG NH2; ASN ND2; ASP CG; CYS SG; GLN NE2; GLU CD; GLY not included; HIS NE2; ILE CD1; LEU CD1; LYS NZ; MET CE; PHE CZ; PRO CG; SER OG; THR CG2; TRP CH2; TYR CZ; VAL CG2.

### Estimation of Changes in Bond Angles Between the XylE Conformers

The *Φ*−*Ψ* bond angles of all of the side chains in the eight XylE conformers were obtained by processing at the “WHAT IF” web site http://swift.cmbi.ru.nl/servers/html/index.html. Angular displacements of all the conformer side chains were obtained from the square roots of the sum of all angular displacements squared. These were then graphed as shown in Fig. 7b.

### Sequence Analysis

The reviewed section of UniProt (SwissProt) was used to identify classes of protein sequences using SRS-the Sequence Retrieval System (http://srs.ebi.ac.uk/srsbin/cgi-bin/wgetz). This contained 540958 sequence entries, comprising 192206270 amino acids abstracted from 221942 references. SRS allows controlled selection of proteins. Using Boolean logic the various sets of proteins were then examined using the EMBOSS fuzzpro, a (PROSITE style) pattern searching utility (http://emboss.bioinformatics.nl/cgi-bin/emboss/fuzzpro. The pattern employed was phenylalanine or tryptophan [FW] × {2–10}[FW] to identify subsections of each protein sequence between 4 and 12 amino acids long and these were both initiated and terminated by either F or W. A simple Perl script then counted the occurrences of the amino acids A, G, P, V, or C to produce the distributions. The EMBOSS utility ‘Shuffleseq’ generated the randomised sets of data. Similar searches were done in the database for transmembrane proteins TOPDOM. http://topdom.enzim.hu. (Tusnády et al. 2008).

### Transport Model Construction and Simulation

A gated transport model with two sequential binding sites, separated by an external and internal vestibule, situated within a gated channel, was simulated as shown in Figs. 10 and 11. The simulation program employed was Berkeley Madonna http://www.berkeleymadonna.com/ and was similar to that previously described (Cunningham and Naftalin 2013). The new feature introduced here it the two gates. The transport model simulates the salient features of the transport channel as described in this paper: namely a gated vestibule containing a high affinity sugar binding site leading via a narrow gating structure to an internal vestibule containing the lower affinity binding site, bounded by a second gate, leading to the internal bathing solution. Sugar is assumed to bind to the higher affinity site *K*_D_ = 1 and 20 mM to the lower affinity site and to diffuse from the external solution to the first binding site and from the first to the second site via the external/central cavity, where it binds and dissociates and then diffuses onwards. The effect of raised sugar concentrations in the distal vestibules will be to reduce the net rate of sugar movement from the external solution, where for the purposes of this simulation glucose concentration is fixed at 50 mM. The objective of this transport model is to demonstrate the effects of altered rates and chronicity of the sequential gate closures on net sugar transit rates. The gating rates are controlled by a sine wave timer, whose frequency and amplitude are altered at will. The phasic relationships between the gates are altered independently. For this simulation, it is assumed that the gates have the same opening frequency, but can be open and closed simultaneously—in phase (0°), or in antiphase (180°) with gate 1 (Fig. 11c, f). The frequency of ligand association and dissociation rates to and from the binding sites is controlled as follows: *K*_D_ = *k*_2_/*k*_1_; hence *k*_2_ = *K*_D_.*k*_1_. It is assumed that the ligand association rates are the same for both sites. However, the slower diffusion rates of ligands within the external and internal vestibules affect the apparent rates of association and dissociation within the vestibules substantially. Holding *K*_D_ constant and varying *k*_1_ changes both *k*_1_ and *k*_2_ proportionately. The effects of the gating sequences were also investigated extensively with varying gate numbers (2–4) in series with different open and closed timings. The principle finding will be illustrated: namely that opening and closure rates of gating with similar or higher frequencies to ligand dissociation rates do not alter the net ligand transport rate across the network.

## Results and Discussion

### The Affinities in the Central Zone

**Fig. 1:**
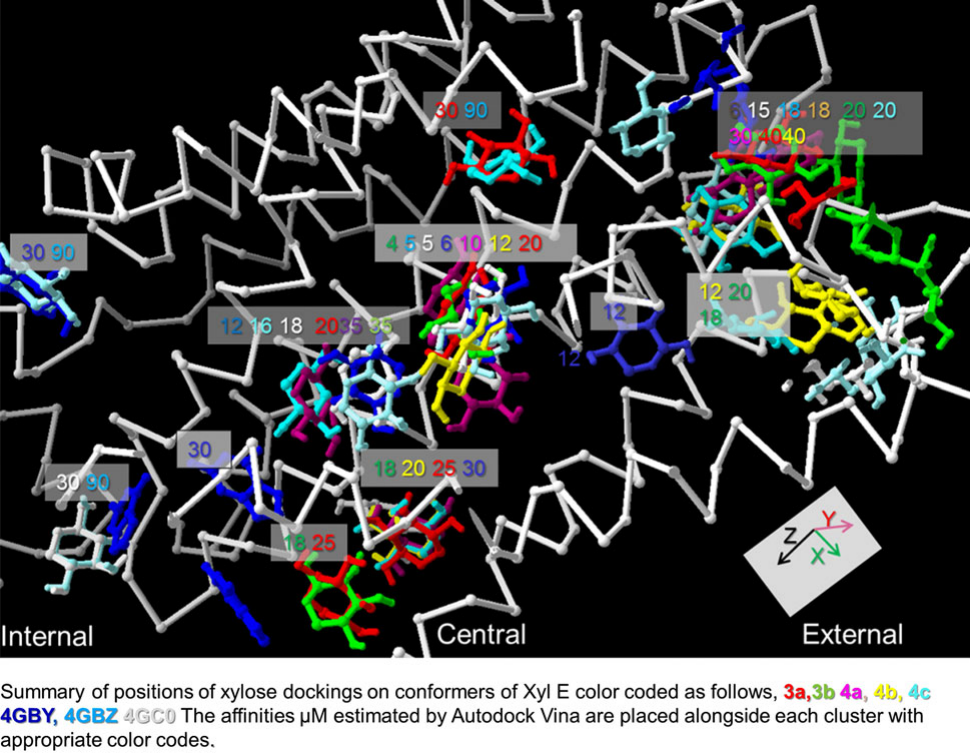
The best-fit superposition of images. As described in the text, there are a number of coincident docking sites represented in all regions of the transporter. Superimposed sets (36) of Autodock Vina dockings in which the nine highest affinities in each run are docked on XylE conformers 3*a,*(*red*), 3*b*, (*green*); 4*a* (*maroon*), 4*b* (*yellow*) and 4*c* (*cyan*) and the outward-facing GBY (*navy blue*), GBZ (*royal blue*) and 4GC0 (*white*). Alongside each group is the estimated range of *K*_*i*_’s of each cluster at that position estimated by Autodock Vina from the estimated Gibbs free energy of binding (Color figure online)

**Fig. 2:**
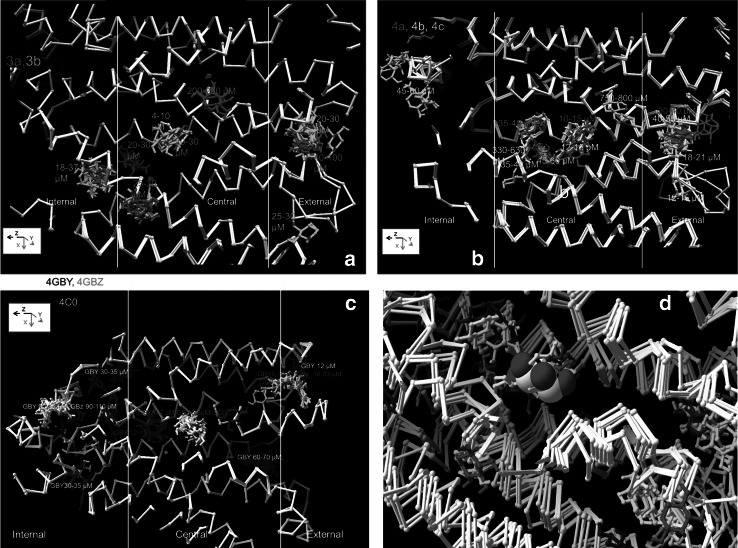
Side views showing xylose docking to **a** conformers 3a and 3b, **b** conformers 4a, 4b and 4c and **c** 4GBY, 4GBZ and 4GC0. The *white vertical lines* subdivide the transporter regions along the *z* axis approximately into external vestibular, central and internal vestibular zones; **d** The crystal docked xylose shown as the 3D rendered CPK coincides with the high affinity docking site as obtained with several XylE conformers shown as *ball* and *stick* forms (Color figure online)

Xylose docking to the eight *XylE* conformers is shown by the following colour codes, PDB (4Ja3a) 3a red, PDB (4Ja3b) 3b green, PDB (4ja4a) 4a mauve, PDB (4ja4b) 4b yellow, PDB (4ja4c) 4c cyan and PDB 4GBY navy blue, PDB 4GBZ light blue and PDB 4GC0 white (Figs. 1, 2). The carbon chains of the various conformers are superimposed in Fig. 1, using the best-fit iterative ‘magic fit’ algorithm supplied by SwissProt Deepview, but only conformer 3a carbon chain is shown, the others are suppressed to give a clearer view. Figure 1 is a composite of thirty-eight Autodock Vina docking runs on all eight XylE conformers. The single highest affinity ligand in each separately identifiable cluster is displayed and the extent of docking surface occupied by each of clusters is shown in Fig. 2. The assigned affinities (see Methods) beside each ligand are colour coded to the appropriate conformer. High affinity docking occurs in the central position in all conformers except 4c. The highest affinity is on the partially occluded inward-facing conformer 3b (4 µM), the next highest is to the outward facing partially occluded conformer 4GBZ (5 µM). Docking also occurs at the two other partially occluded outward-facing conformers 4GBY and 4GC0 and the three inward-facing apo and holo-conformers 4a, 4b and 3a. The consistency of the docking patterns between the conformers attests to the reliability to the docking procedure and the existence of peripheral lower affinity sites in addition to the central site.

No significant difference exists between highest affinities at the central binding site of the outward 4GBY (6 µM) 4GBZ (5 µM) and 4GC0 (5 µM) and the inward conformers (3b 4; 4a 10; 4b 12; 4c 5 µM), (Figs. 1, 2). Some poses adopted by ligands lie across the central narrow zone. This implies that xylose can change its posture within the docking site so that it can eventually adopt a pose which permits it to slide through the narrow aperture between the internal and external vestibules. This is particularly the case with hexose and pentose sugars, such as d-glucose and d-xylose, where puckering of the pyranose ring structure between chair and boat conformations can alter the maximal ring diameters between for O5–O3 distances from 4.14 Å chair to 3.8 Å boat i.e. by Δ 0.34 Å O5–O4 chair 3.68 Å to O5–O4 boat 1.55 Å i.e. Δ 2.13 Å and for O5–O2 chair 3.68–3.42 Å i.e. Δ 0.26 Å, as we have measured directly in our docking studies. Thus assuming that the sugar can adopt a pose which minimises its radius, it is apparent that the boat shaped xylo-pyranose ring has a minimal radius of approximately 1.9 Å (Barnett and Naidoo 2010).

### Comparison of Docking Between the Outward and Inward Conformations

The conventional single alternating site model predicts that xylose should dock exclusively to a central site in both the open-outward conformation and the inward-facing conformations. Docking at other sites might be expected to occur with lower affinity at the inner vestibular sites in the outward-facing conformers 4GBY, 4GBZ and 4GC0 and lower affinity at sites within the external vestibule in the inward-facing conformers 3a, 3b and 4a, 4b, 4c. However xylose dockings with moderately high affinity are observed at several shared sites at all the inward and outward-facing conformations, (Figs. 1, 2).

Moderately high affinity sites are also present at the external surface of 3a, 3b; 4b and 4c and 4GBZ, GBY and 4GC0, suggesting that sugar from the external solution is first encountered and captured at external surfaces of the transporter. Ligand flow towards the central site may be promoted by its higher affinity. These docking patterns do not fit with the expectations of the Jardetzky alternating access paradigm (Jardetzky 1966; Henderson and Baldwin 2013).

### Comparison of Docking Between Liganded and Unliganded Conformations

The induced-fit theory of enzyme catalysis suggests that ligand–protein interaction initiates protein conformational changes e.g. hexokinase, that increase the affinity of ligand binding (Kuser et al. 2000). Similar induced-fit mechanisms have been deduced for transporter action, based on the absence of crystallographic evidence of a sugar bind site of ligand binding site to the apo form of LacY transporter (Mirza et al. 2006). Autodock Vina has no capacity to alter the conformation of the protein to which ligands are docked. Thus the expectation of the alternating access model would be that affinity of xylose docking in silico to XylE should be much higher towards the occluded liganded or inward-facing halo-conformations of 3a and 3b, than towards the unliganded apo inward-facing conformations, 4a, 4b and 4c (Quistgaard et al. 2013). This model prediction is not confirmed by the docking studies. The highest estimated xylose docking affinity on open inward-facing conformer 3a is 20 µM, whereas the highest affinity of xylose docking to the other open inward conformer 3b is 4 µM. The highest xylose affinities to the inward-facing apo-conformers 4a and 4b are 10 and 12 µM. Thus the affinity of xylose to the unliganded open inward conformers is intermediate between the docking affinities at the central region of the holo-inward conformers. Accordingly, there is no apparent induced-fit adaptation of the central binding region to the docked ligand. The central docking site affinities of the halo open-outward forms 4GBY, 4GBZ and 4GC0 are all similarly high (5–6 µM). The affinities of xylose to the unliganded form are intermediate between the widely different affinities of xylose at the central docking region of the two inward-facing liganded forms. The main conclusion from these findings is that adaptive conformational changes do not appear to be a necessity at the central site for ligand binding to occur. This indicates that XylE, unlike LacY (Kaback et al. 2011) permits ligand binding to the inward-facing apo and holo-forms of the transporter with comparable binding affinities and therefore xylose docking to XylE conformers does not entirely corroborate the induced-fit model. However, as will be shown below, there is other clear evidence suggesting that ligand docking does induce small conformational changes.

### Large Area Sites may Permit Simultaneous Binding of Two Ligands

Several sites can accommodate more than one ligand; two of these large sites are in the internal vestibule, one in central zone and two in the external vestibule (Figs. 1, 2). Similar docking patterns at vestibular sites have been reported previously with glucose binding to GLUT1 (Cunningham and Naftalin 2013). Sites where ligands bind simultaneously could permit accelerated exchange (Naftalin and De Felice 2012; Naftalin 2010). The large xylose binding site in the upper part of the external vestibule in Fig. 1 has a wider affinity range and is present in all eight conformers, whereas docking to the lower vestibular site only occurs on the two inward 3b, 4c and two outward-facing conformers 4GBZ and 4GC0, but not to 3a, 4a, 4b or 4GBY.

It could be argued that ligand docking within the external vestibule is uncertain since it occurs in regions where in the case of the inward-facing isoforms at least, 3a, 3b 4a, 4b and 4c, some of the linker regions are unstructured (see supplementary Fig. 2). However, there is some coincidence with ligands docking on the outward-facing conformers, 4GZ and 4GC0 in the lower sector cluster docking site and the docking site in the sector occurs in all three of the outward-facing conformers 4GY, 4GZ and 4GC0. Since these latter conformers have fully defined crystal structures, this provides some validation for the docking site positions assignments in the five inward-facing conformers.

The extent of unstructured sequences is much less in the inner vestibule than in the outer vestibule and affects none of the ligand binding clusters in this region. Some variability in the external vestibular volume may be ascribable to the fact that the five inward-facing conformers contain two unstructured loop sequences F304–A314 and L435–H440 (Supplement Fig. 2). This lack of definition results from positional variability in these external linkers when in the open-out conformation and is also mirrored by the large RMSD observed in these regions Fig. 4a. It should be noted that none of the unstructured sequences fall within the TM regions mapped in Fig. 4b.

### The Relationship Between Internal and External Vestibular Volumes in XylE Conformers

**Fig. 3:**
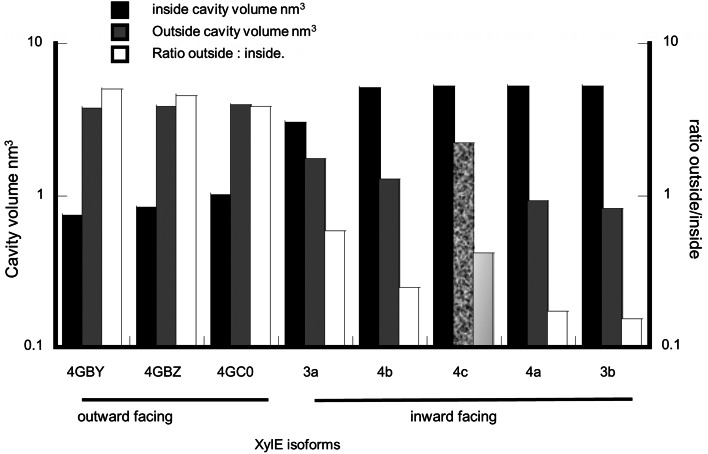
Histogram showing the size of the inside tunnels nm^3^ plotted in order of increasing inside cavity volume. The volume of the corresponding external tunnels and the ratio of external: internal are also plotted. There is a general decrease in the ratio of external: internal volume as the internal volume increases: however, exceptionally the external vestibular tunnel volume is larger than would be predicted if there was a rigidly controlled inverse relationship between internal and external vestibules

The external and internal vestibular sizes vary greatly. The open-inwards conformers have average inward-facing vestibular cavity volumes of 4.81 ± 0.45 nm^3^ and average outward-facing cavity volumes of 1.41 ± 0.27 nm^3^. In contrast in the open-outward conformation, the average inward-facing vestibular space is 0.87 ± 0.08 nm^3^, whereas the average outward-facing space is 3.8 ± 0.07 nm^3^ (Fig. 3 and supplement Fig. 1). The inward/outward vestibular volume ratio of inward-facing conformers is (4.1 ± 0.92); whereas this ratio averages 0.23 ± 0.02 (*P* < 0.001) in outward-facing conformers; an overall change of 18.0 ± 4.3–fold (*P* < 0.001). At first sight there appears to be an inverse correlation between the volume of the inside-facing vestibular space and outwards-facing spaces. However, the apo-conformer 4c is exceptional because it has both a large internal vestibule (5.3 nm^3^) and a large external vestibule (2.2 nm^3^) (shaded column, Fig. 3), approaching that of the outward-facing holo-conformers 4GBY, 4GBZ and 4GC0, with average volumes 3.8 nm^3^. Thus the apparent inverse relationship between the inward and outward-facing transporter conformations is not as rigid as the Jardetzky rocker-switch model implies (Jardetzky 1966; Henderson and Baldwin 2013). This irregularity in the outward: inward vestibular volume ratio illustrates that transmembrane carbon chains have some capacity for independent motion to permit such an extensive variability.

### Deviations Between XylE Conformers in the Transmembrane Helical C-α Positions

**Fig. 4:**
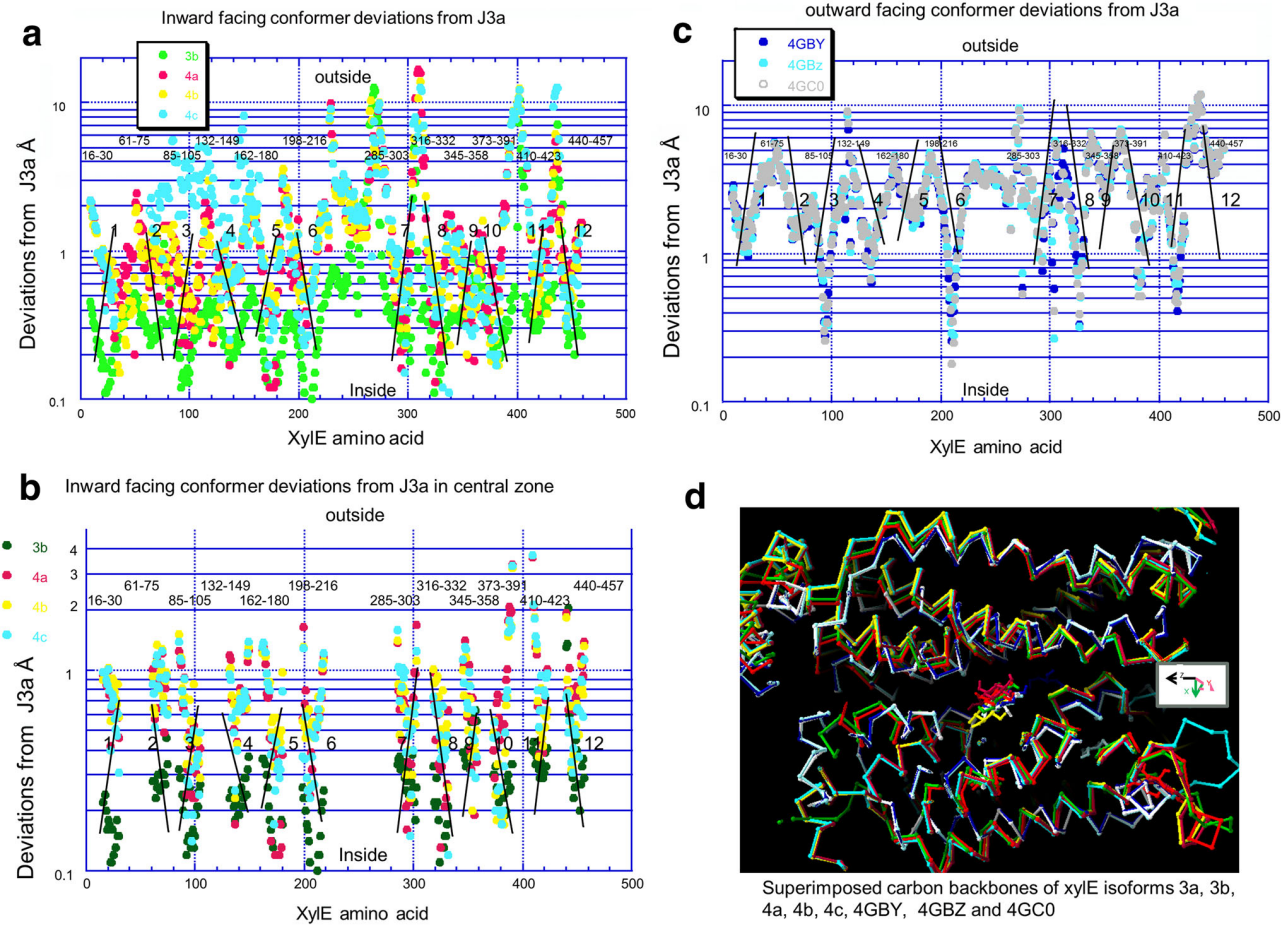
Histograms showing the RMS deviations in the Cα positions of XylE conformer 3D structures as obtained from the PDB files. These are all referenced to the inward-facing holo-conformer 3a. The same *colour* code is used as in Figs. 1 and 2 to signify each conformer. **a** shows all the RMSDs of conformers 3b, 4a,4b and 4c. **b** shows the RMSD of the Cα position of amino acids with the transmembrane helical regions. **c** shows the RMSD of the Cα positions of amino acids in outward-facing conformers 4GBY, 4GBZ and 4GC0. **d** Superimposed carbon backbones of XylE conformers *colour* coded as in Fig. 1 showing 3a, 3b, 4a, 4b, 4c, 4GBY, 4GBZ and 4GC0. The ligands binding to the high affinity central docking site are also shown. The divergence of the extramembranous strands is much more obvious than in the central region. See also Fig. S1 (Color figure online)

The carbon chain mobility of XylE may be quantitatively gauged by comparing the changes in relative position of the individual carbon atoms between conformers, as assessed by the RMS deviation (RMSD) (see Methods). The relative movements of all the C-α positions are referenced with respect to the inward-facing holo-conformer 3a and illustrated by mapping the relative positions along the length of the protein chain, Fig. 4d. As expected, larger position changes occur in the unsupported N and C terminals and between external and internal linkers regions between the transmembrane helices (TMs), than within the TM’s (Fig. 4a). This is shown by the larger excursions between the inwardly upwards directed and outwardly downward directed diagonals which cover the TMs regions as identified by the 3D crystal structures. The deviations in linker chains are largest in inward-facing apo-conformers 4a, 4b and 4c

**Fig. 5:**
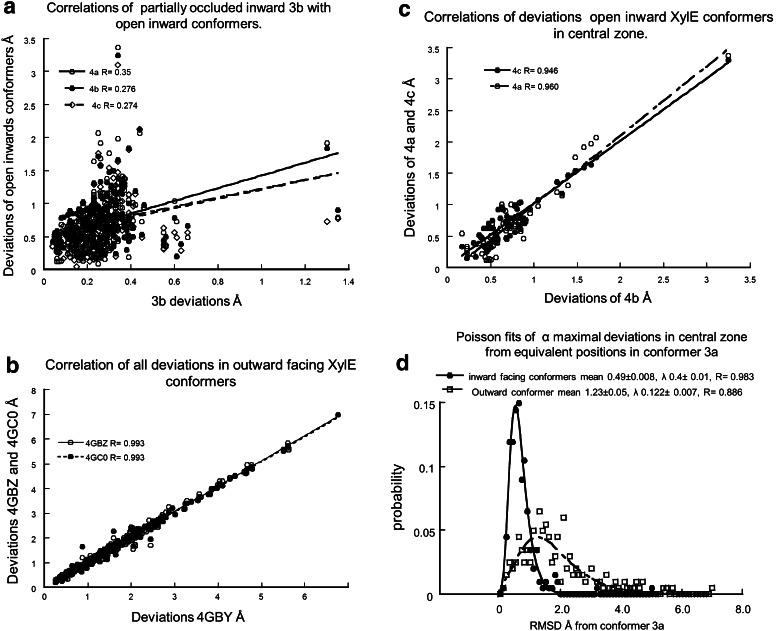
Linear regression lines between the equivalent Cα positions in the central transmembrane helical amino acids. **a** Regression lines of the deviations of inward-facing holo-conformer, 3b and inward-facing apo-conformers 4a, 4b and 4c. **b** Linear regression lines between the Cα positions of the holo outward-facing conformers, 4GBY and 4GBZ and 4GC0. **c** Linear regression line between the inward-facing apo-conformers 4b and 4a and 4c. **d** Poisson distributions and fits to the deviations of Cα positions of the inward-facing holo-conformer 3b, with the inward-facing apo-conformers 4a, 4b and 4c (*filled circles*) and outward-facing conformers 4GBY, 4GBZ and 4GC0 (*open squares*)

The average dispersion between the carbon chain atoms of the four inward-facing conformers (3b, 4a, 4b and 4c) (10.74 ± 2.26 Å SEM.) exceeds that of the three outward-facing holo-conformers (3.62 ± 0.45 Å, *P* < 0.0001). This point is quantitatively illustrated by the higher regression coefficients the C-α positions between each of the outward-facing conformers 4GBZ and 4GC0 with 4GBY (*R* = 0.993) (Fig. 5a) than between the three open inward-facing conformers 4a (*R* = 0.96) and 4c (*R* = 0.946) and with 4b (Fig. 5c).

Additionally, the maximal deviations between the C-α positions of the open inward-facing 3a, 3b, 4a, 4b and 4c conformers, in the central TM region are much larger (5.17 ± 1.17 Å) than between the outward-facing conformers 4GBY, 4GBZ and 4GC0 (1.54 ± 0.13 Å) (*P* < 0.001) (Fig. 5a–c). The central zone mobility of the three open inward-facing apo-conformers 4a, 4b and 4c (6.33 ± 0.08 Å) greatly exceeds that of all five partially occluded inward or outward-facing conformers 3a, 3b, 4GBY, 4GBZ and 4GC0 (1.67 ± 0.03 Å, *P* < 0.001). This indicates that in the apo condition, regions of the central TM helical chains have similarly high mobility to those in unsupported extramembranous regions. The presence of the centrally bound ligand locally reduces the carbon chain mobility. A similar observation has been made using molecular dynamic simulations to monitor the fluctuations the C-α atoms with regard to GLUT4 (Mohan et al. 2010).

The distribution patterns of maximal deviations of the C-α atoms in the central zone of the inward-facing and outward-facing conformers fit Poisson distributions (correlation coefficient *R* = 0.983, for the Poisson fit to the inward-facing deviations of 3b, 4a, 4b and 4c and *R* = 0.866 for fit to the C-α atom deviations of the outward-facing conformers 4GBY, 4GBZ and 4GC0). The mean deviation of the inward-facing dispersion is 0.49 Å and *λ* = 0.4 Å; whereas the distribution of the outward-facing C-α atoms deviation from the equivalent positions in the inward-facing conformer 3a has a much wider dispersion, mean 1.23 Å and *λ* = 0.12 Å (Fig. 5d).

### Deviations in Amino Acid Side Chains Between Conformers in the Regions Close to the Central Binding Site

**Fig. 6:**
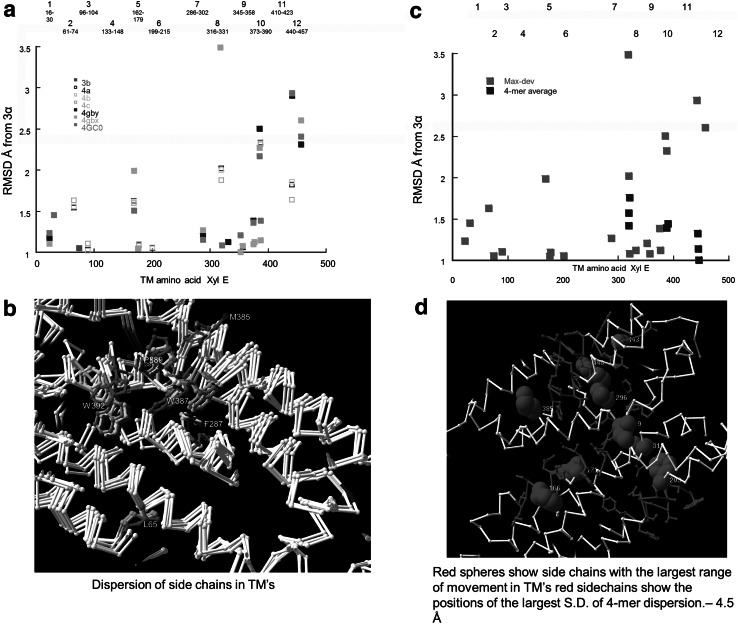
**a** Map showing large RMSD deviations of C-α positions in TM’s of XylE conformers. The map of the main C-a deviations shows that the major deviations occur in TMS 7–12. The main changes occurring in TMs 10–12 in conformers 4a, 4c, 4Gbx 4Gby and 4GC0 in TM 10 and the outside-facing holo-conformers 4Gbx 4Gby and 4GC0 in TM12. **b** Superimposed XylE conformers showing side chain divergences. See also Table 2 for the changes. The large changes in P389 and W392 suggest a possible gating structure. Additionally, the large changes in F287 and W387 indicate a second gating position. **c** Map showing maximal deviations of C-a positions from those in J3a. *Red* shows the C-α maximal deviations of individual amino acids. *Blue squares* show the 4-mer average deviations. Maximal deviations occur in TMs 7 9 and 11. **d** Positional changes in XylE 3-D structure showing 4-mer regions of maximal deviation within the C-a within the TMs. The *red spheres* show the largest SD of side chain deviations within the TM regions. Note almost the entire central channel shows large divergences between conformers and there are regions at either end with large side chain SD (Color figure online)

Comparison of the position changes in all the side chains was performed by measuring the relative positions of all the most distal side chain atoms in all conformers relative to those in conformer J3a (See Methods). This survey shows that some side chains close to the central ligand binding site undergo larger position changes on switching between the various inward-facing conformers, than on switching between the inward and outward-facing conformers. Some of the conformational changes between inward and outward -facing conformers i.e. between 3a and 4GBy, or GBz or 4G0 are smaller than the “thermally”-induced changes between the two inward-facing holo-conformers 3a and 3b and three apo-conformers of the inward-facing conformers 4a, 4b or 4c, Fig. 6a.

In particular the indole groups of centrally positioned W387 and W392, in the inward-facing conformers 3a (red) and 4c (cyan) are twice as widely displaced (9 Å) as between the inward (red) and outward-facing conformers 4GC0 (grey) 3.7 Å (Fig. 6a, b). The deviations in P389 between the outward-facing 4GC0 conformer (white) 4.3 Å and the inward facing 3a (red) are also twice as large as those between the two inward-facing conformers, 4c (cyan) and 3a (red). These conformational changes are not uniform throughout the central zone; much smaller inter-conformer displacements occur between more proximally positioned F173, F24, D27 and T28.

Although important, estimates of displacements of individual amino acid side chains are insufficient to indicate the mobility of local regions within the transmembrane helices (Fig. 6a). By mapping the running 4-mer averages of the maximal deviations of side chain positions between the eight conformers, estimates of the mobility of each coil of the TM’s can be obtained. These are shown in Fig. 6c (blue squares). The regions with high average displacements unsurprisingly coincide with individual amino acids with high C-α chain mobility (Figs. 4b, 6a and Table 1). Only three TM regions have 4-mer displacements that exceed 1.0 Å. These are in TMs 8, where Q317 deviates by 3.5 Å and I319 by 2.0 Å. In TM 10 M385 deviates by 2.5 Å, W387 by 2.36 Å and TM 11 N441 by 2.94 Å. TMs 8 and 10 lie adjacent to each other and to the central cavity, whereas TM 11 is positioned on the opposite side of the central cavity.

**Table 1:** Largest positional changes of C-α atoms in central TM region of XylE conformers

XylE AA	Conformer type	Distance Å from 3a Cα
L22	4GC0	1.24
L65	4a,4b,4c	1.54–1.63
Q168	4a,4b,4c,4Gby,4Gbz,4GC0	1.5–2.0
F287	4GbY,4Gbz	1.27
Q317	4GBz	3.49
I319	4c	1.58
L374	4a,4b,4c,4Gby, 4GC0	1.36–1.37
M385	4Gbz,4GC0	2.2
W387	4a,4b,4c;4Gby,4Gbz,4GC0	1.4
N441	4a,4b,4c; 4Gby,4Gbz,4GC0	1.64–1.86; 2.9–2.95
L457	4Gby,4Gbz,4GC0	2.3–2.9

**Fig. 7:**
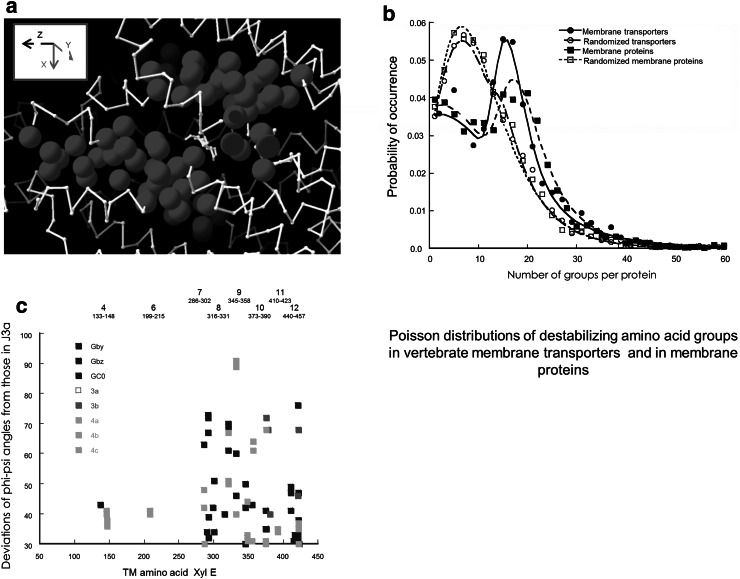
**a** The sequences close to the central high affinity binding site containing a high density of destabilizing amino acids; namely G, A, P, C, S, I and V. The closest proximity of the highest densities of these groups was estimated and plotted as running averaged of ten amino acids, along the entire sequence. This was added to a scaled B-Factor and the data mapped onto a 3D XylE crystal structure. **b** Graphs showing double Poisson distribution fits to the probability of the occurrence of destabilizing groups within transmembrane proteins. The graphs show the probability of occurrence of destabilizing groups in 7231 vertebrate transmembrane transporters (*filled circles*) 109486 groups and 174257 vertebrate transmembrane proteins that are not transporters (*filled squares*) 174257 groups, randomised transporters *open circles*, and randomised proteins (*open squares*). The *lines* show the occurrence probability distributions of the numbers of these destabilizing groups. These are fitted, using a Marquardt–Levenberg algorithm to the double Poisson distribution: S.*λ* ^1^.exp{−*λ* ^1^.(*X*−μ^1^)−exp(−*λ* ^1^. (*X*−μ^1^)} + (1−S).*λ* ^2^.exp{−*λ* ^2^.(*X*−μ^2^)−exp(−*λ* ^2^.(*X*−μ^2^)}. Where *λ* ^1,2^ are the variances in the two subpopulations, μ^1,2^; the mean values *X* are the numbers of the sub-population groups per protein and *S* is a scaling factor. *S* adjusts the relative contribution of the two subpopulations to the overall occurrence probability. It is evident that randomization of the amino acid sequences reduces the modal frequency of occurrence of destabilizing groups in both transmembrane transporter proteins from 16.04 ± 0.31 to 7.08 ± 0.25 (*P* < 0.001). A similar reduction occurs with transmembrane proteins that are not assigned within the database as transporters. On randomising the protein sequence, there is a significant reduction in the scaling factor *S* from 0.46 ± 0.07 to 0.00 ± 0.03 (*P* < 0.001). This indicates that there is a non-random element to the occurrence of destabilizing sequences within transmembrane proteins whether transporters or not. **c** Map showing the positions of major deviations in combined *Φ*−*Ψ* *angles* of XylE conformers in TMs in comparison to inside-facing holo-conformer J3a. The outside-facing holo-conformers Gby, Gbz and 4GC0 are all shown as *navy blue squares*; the deviation of inside-facing holo-conformer from 3b is shown in *red* and the apo inside-facing conformers are shown as *green squares*. It can be seen that the major changes are mainly in TMs 7–12. The holo-apo transformations occur mainly in Tm- 7–10 whereas inside to outside-facing transformation of the holo-forms mainly represents increases in TMs 7–9 and 12. Note that large deviations occur between the two inward-facing holo-forms in TMs 10 and 12 (Color figure online)

The *Φ*−*Ψ* angular displacements of the side chains have also been examined (Fig. 7c). The largest angular displacements from J3a are in TM’s 7–12. Large displacements occur between the two inward-facing holo-conformers in TM’s 10 and 12 (red squares) and between the inward and outward-facing holo-conformers in TM’s 7–10 (blue squares). A smaller displacement also exists between the apo and holo inward-facing conformers in TMs 4 and 6. There is a large cis–trans switch between the inward-facing holo and apo-forms of P389 (Figs. 6b, 7c). The dihedral *ψ* angles of the apo-conformer are similar to the holo-forms −88.0° whereas the *φ* angles changes by +72° to +22.2° on changing from inward-facing apo to holo conformation. The P389 conformation changes little between the various semi-occluded conformers 3a, 3b 4GBY, 4GBZ and 4GC0.

### Examination of the Causes of Central Zone Instability

The disruptive effect of proline and glycine residues previously has been discussed in relation to the flexibility of the transmembrane helical regions of GLUTS (Williams and Deber 1991; Sansom 1992; Tamori et al. 1994). Many studies suggest that adjacent proline and glycine residues disrupt the stability of transmembrane helical structures (Williams and Deber 1991; Craveur et al. 2013). Primary sequences containing high densities of glycine, alanine, serine, cysteine, proline, valine and isoleucine are known to have reduced α-helical stability and this is likely to produce small-scale movements within the protein chains- higher reptation rates (Langosch and Arkin 2009). Proline induces kinks in alpha helices by its lack of H-bonding capacity. This permits increased mobility of the carbonyl group three-four residues removed, hence introducing helical instability.

The unstable amino acid sequences within 5 Å of the high affinity central docking site are detected by combining a scaled B-factor, or temperature factor obtained from the PDB files, indicative of thermally activated atomic motion with a similarly scaled density function of the destabilizing amino acids. This map includes a sequence of five amino acids G388, P389, V390, C391, W392, containing four destabilizing amino acids G, P, V and C that match all the criteria this motif is a source of carbon skeleton instability (Fig. 7a) (Langosch and Arkin 2009). In Fig. 4b this motif 388–392 is shown to have the highest deviation from conformer 3a. The average deviation in the inward-facing conformers is 2.75 ± 0.38 Å for the outward-facing conformers and this motif deviates from 3a by 5.3 ± 0.38 Å.

A search into the incidence of occurrence of this GPVC motifs bracketed by tryptophan or phenylalanine residues in the SwissProt protein structure database shows that they occur in 95 % of all transporter proteins, including GLUT1. A further search for vertebrate membrane transporters identified 8022 proteins. These proteins contain 122724 G-P-V-C patterns = 14.7 patterns per protein (Fig. 7b). There are 5305 vertebrate transmembrane proteins WITHOUT the keyword *transporter*; these have 35769 motifs = 6.05 per protein. A similar search was made in the database of membrane proteins TOPDOM (Tusnády et al. 2008) and revealed 39022 motifs containing G-P-V-C patterns in approximately 6,000 transmembrane proteins. The high frequency of these disruptive motifs in transmembrane helical proteins is well recognised (Brandl and Deber 1986).

The relatively large dispersion of these central positioned disruptive motifs between the eight conformers particularly in the apo-conformer forms suggests that the TM flexibility is reduced locally by ligand binding. Similar deductions have been made on the basis of molecular dynamic studies of glucose binding to a homology model for GLUT4 (Mohan et al. 2010). It is evident that both vertebrate transmembrane proteins and transmembrane transporter proteins are over-represented in the frequency of occurrence of G-P-V-C patterns bracketed by either tryptophan or phenylalanine separated by between two and ten amino acids 14–17 per protein. Although over-represented in the range 14–17 occurrences per protein there is under representation in the range 4–10 groups per protein (Fig. 7b). Randomization of the amino acid sequences within the each protein changes the pattern’s distribution from a double to a single Poisson distribution. The goodness of fit of the double Poisson distribution *R* = 0.97 and for the single Poisson distribution of the randomised sequences *R* = 0.99. These findings suggest that evolutionary pressure may have increased the frequency of destabilizing sequences of amino acids within transmembrane proteins.

### The Relationship Between Tunnels, Cavities and Docking Sites in XylE

**Fig. 8:**
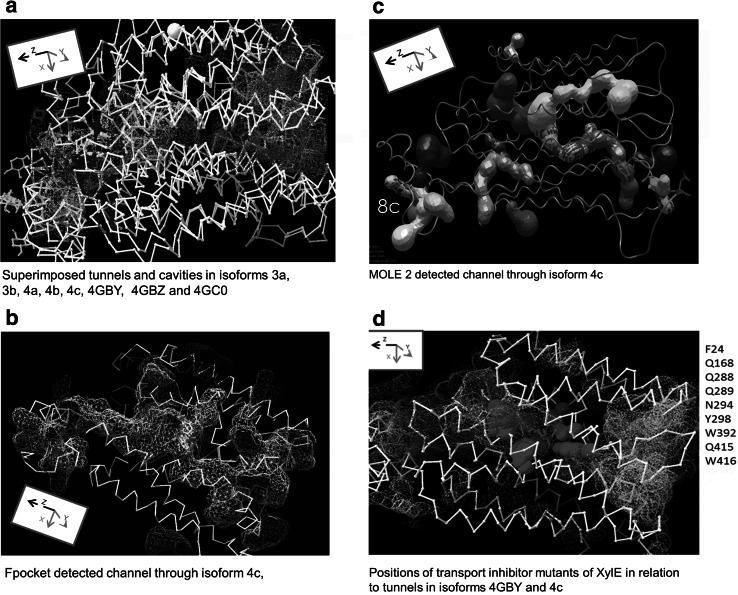
**a** Views of the superimposed large tunnels mapped as a mesh of varying *colours* using Swissprot Deepview’s roller ball method of all eight of the available conformers of XylE. The estimated total volumes of the tunnels at the internal surface and external tunnels in nm^3^ are shown beside each panel. The tunnel maps are shown in the *bottom right panel*. The nested tunnels generate a fully spanning channel through the transporter. **b** Fpocket derived image showing a slab view of the fully open penetrating tunnel shown as a *yellow mesh*, through XylE conformer 4c. There is a narrow gap just distal to a docked cluster in the central region of the transporter forming an isthmus between the external and internal vestibular cavities. **c** Mole 2 derived tunnel XylE conformer 4C. The tunnel in 4c completely spans the transporter; the width at its *narrowest point* has a radius of 1.2 Å. The probe radius used with the tunnel-finding MOLE 2 for the tunnels illustrated in Fig. 8c and in supplementary figures was 3.5 Å The radius of the *bottleneck* position at a distance of 8–9 Å from the tunnel opening is 1.2 Å this is close to F290, V422, W 448, M451. The *second bottleneck* with a radius of 1.25 Å occurs close to L172 and L326 and the third with a similar small radius close to W445 and M356. It should be noted that all these amino acids have large side chains where altered rotamer positions can easily alter the pore radii. It can be seen in Fig. 6a and c that these choke points are coincide with regions of high mobility. **d** shows the positions of the transport mutants in XylE (Sun et al. 2012) as red spheres overlaid on the superimposed tunnels of 3a and 4GBY and enumerated at the side (Color figure online)

A primary aim of this paper is to demonstrate by comparison of the eight the static XylE crystal conformers the considerable mobility within the TM region surrounding central high affinity binding zone. The positional changes in the C-α chains and side chain demonstrate the range of movements obtainable. Unlike molecular dynamic simulations these static positions require no further assumptions about the physical forces that generate such changes (Law et al. 2005; Shaw et al. 2010). The cavities and tunnels within a transporter, as with enzymes (Elmabrouk et al. 2011) are signposts to possible routes through which ligands may flow. As already mentioned, using steered molecular dynamics, a tunnel for glucose passage via GLUT4 has been described (Sheena et al. 2011). Also a GLUT1 mutant blocks an external branch of the tunnel network (Cunningham and Naftalin 2013), thereby producing a temperature sensitive transport mutant. Tunnels are demonstrated within XylE using the roller ball algorithm employed by Swissprot Deepview version 4.1, and the Voronoi tessellation algorithm used by ‘fpocket’ and Mole 2 (Le Guilloux et al. 2009; Sehnal et al. 2013). All the xylose docking sites, lie within these tunnels or cavities. Tunnels from the internal surface of the inwards directed conformers (3a, 3b and 4a, 4b, 4c) penetrate more deeply into the core than the tunnels from the external surface, so have larger volumes, (supplement Fig. 1). Conversely, tunnels from the external surface of the open-outward conformation, 4GBY, 4GBZ and 4GC0 extend more deeply towards the protein core than those originating from the internal surface. The gaps between the tunnel ends, in conformers 3a, 3b and 4a, 4b, 4c, are almost filled by cavities in the intervening space. Superposition of conformers 4a, 4b, 4c and 4GBY, demonstrates a continuous tunnel traversing the entire core of the transporter, as shown with SwissProt Deepview roller ball tunnel-finding software. The fusions between the tunnels and cavities are attainable by conformer inter-conversions (Fig. 8a and supplement Fig. 1). Thus condensation of tunnels with cavities produces an ephemeral channel that could facilitate ligand transit to the alternate side of the protein. Two branches in the external vestibule coalesce in the central region coincident with the high affinity binding site. The tunnel then extends through a narrow aperture radius 1.2 Å into the internal vestibule, where it opens to the internal surface, is detectible with both Fpocket and MOLE 2 (Fig. 8b, c; Tables 1, 2). The tunnel image produced by Mole 2 in the inward apo-conformer 4c demonstrates tunnel continuity across the entire transporter. None of the other conformers of XylE has such extensive tunnel networks; the closest to complete channel formation being in conformers 3a and 3b. The probe radius used with the tunnel-finding MOLE 2 for the tunnels illustrated in Fig. 8c and in supplementary figures was 3.5 Å. The radius of the bottleneck position at a distance of 8–9 Å from the tunnel opening is 1.2 Å; this is close to F290, V422, W448, M451. The second bottleneck, with a radius of 1.25 Å, occurs close to L172 and L326 and the third, with a similar small radius, close to W445 and M356. It should be noted that all these amino acids have large side chains, where altered rotamer positions can easily change the pore radii. It can be seen in Fig. 6a and c that these choke points coincide with regions of high mobility. It is evident that in the bottleneck regions the radius will have to expand by 0.7 Å to accommodate the minimal xylose ring diameter of 3.8 Å.

**Table 2:** Amino acid positional changes in central channel neck region RMS Å

Conformer	W298–L65	Q168–W392	W392–I171	F24–N117	F24–I171	F24–W416
3a	5.8	3.38	3.45	7.46	4.48	3.56
3b	5.59	3.38	3.45	6.10 (1.36)	4.49	3.56
4a	5.02	8.38	10.81	6.48	3.71 (0.78)	3.82 (0.3)
4b	5.08	8.35	10.8	6.52	4.82	3.83
4c	5.04	8.48 (5.1)	10.92 (7.47)	6.52 (0.98)	3.82	3.8
4Gby	5.07	3.8	9.46	6.68	6.68 (2.97)	3.64
4GBz	4.77	4.93	12.73 (3.3)	6.41	6.41	3.68
4GC0	5.36 (0.6)	3.8 (4.7) (1.13)	10.75 (1.98)	5.39 (1.29)	5.39 (1.29)	3.46 (0.37)

Recently, it has been proposed that allopurinol and xanthine gain access to the central zone from the cytoplasmic surface of the fungal *Aspergillus Nidulans* uric acid UapA transporter via a tunnel lined with hydrophilic residues (Diallinas 2013).

### Coincidence of Transport Mutant Sites and Internal Tunnels

Sun et al. (2012) have uncovered a number of XylE transport mutants: F24A, R133C/H/L, Q168A, Q288A, N294A, Y298A, R341W, W392A, Q415A and W416A. These sites are mapped as red spheres close to the central xylose docking site (red) are shown in Fig. 8d. The transport inhibitor mutants are situated in the same central region close to the central docking site as the reputation sites discussed above (Fig. 6a). These mutants may interfere with ligand flow, either by interrupting flow along the intermolecular tunnels, as discussed previously (Cunningham and Naftalin 2013), or by increasing the stability of unstable regions, as with the G-P-V-C motif.

### A Transport Model Based on the Multiple Docking Sites within Evanescent Intramolecular Tunnels

**Fig. 9:**
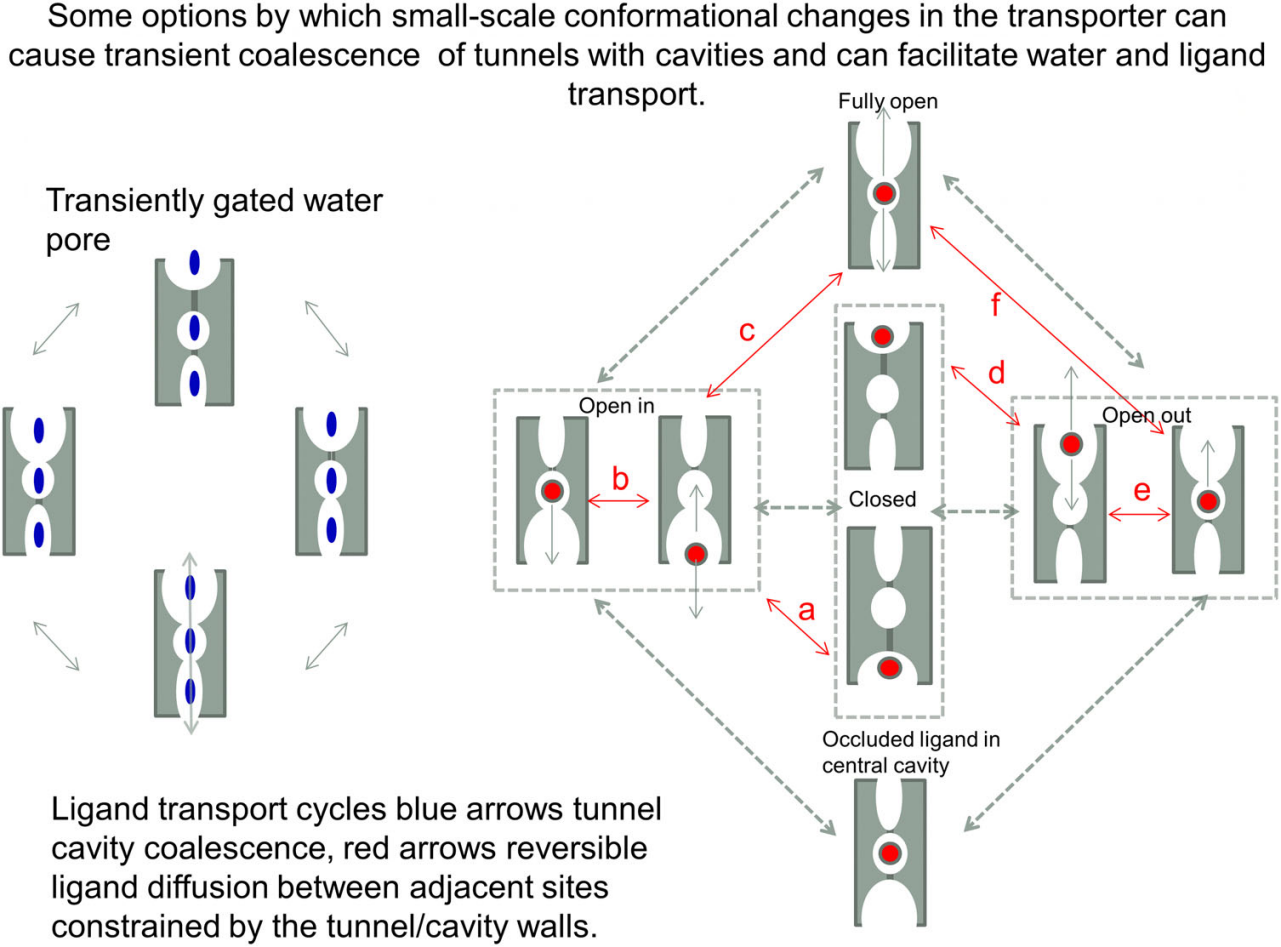
Diagram showing how coalescence of tunnels with cavities with transporter conformational changes can increase osmotic permeability and ligand permeability

**Fig. 10:**
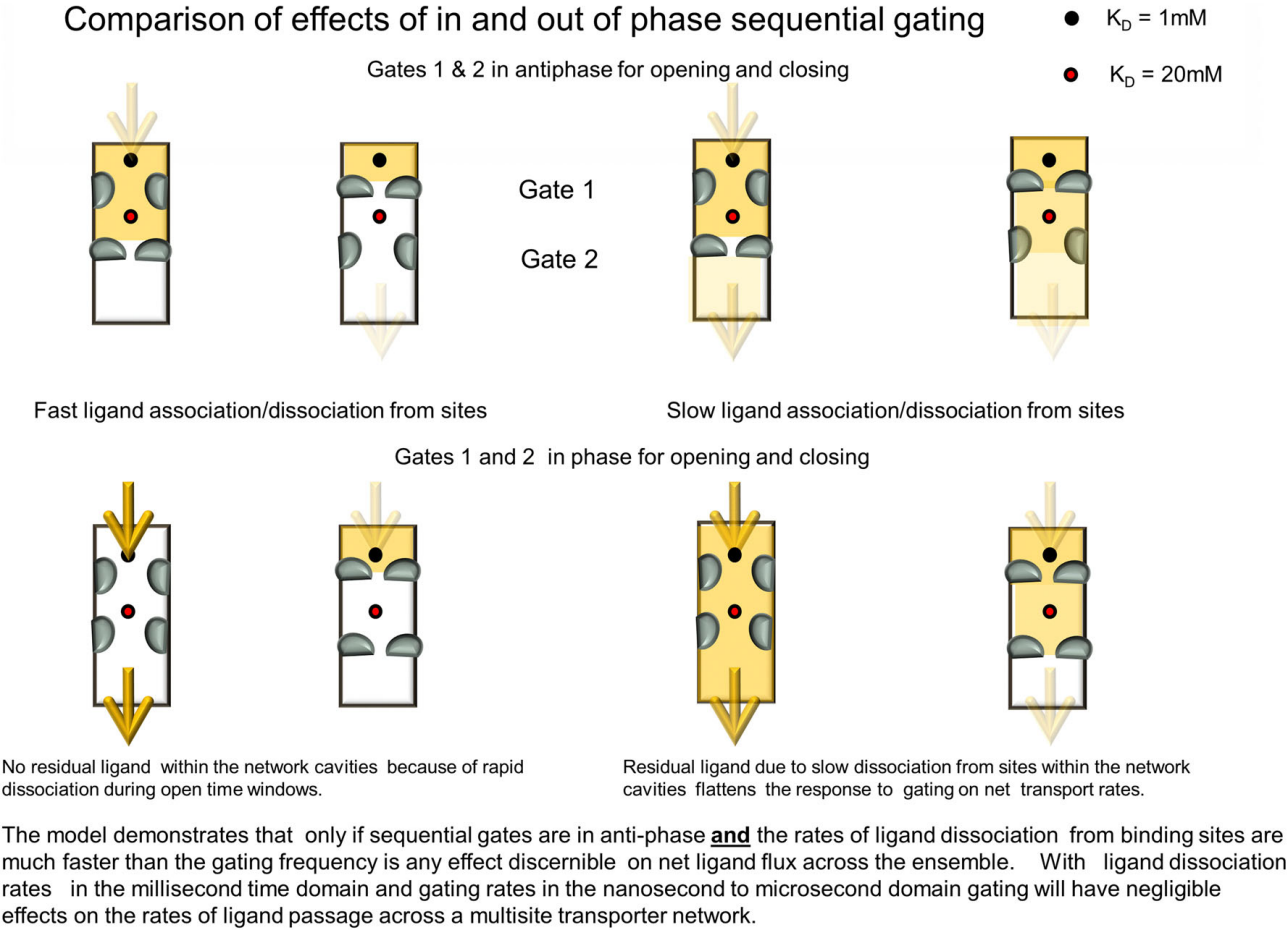
Cartoon showing the effects of in phase and antiphase gating on ligand transit across a two site transporter. The effects of slower rates of ligand association/dissociation from the sites are illustrated by the *colour* filling the vestibules during the gating *open* and *closed* intervals (Color figure online)

The tunnel images in Fig. 8 and Fig. S1 evoke a transport mechanism incorporating elements of both the alternating access and the fixed multisite models (Naftalin and De Felice 2012; Naftalin 2010; De Zutter et al. 2013) Short-range motions of the TMs can lead either to tunnel coalescence with pre-existing cavities, or budding of cavities from the tunnels as they lengthen or shorten. Coalescence between opposing tunnels will ultimately generate a channel that may transiently span the transporter. This suggests that the ligand trajectory occurs as a series of independent steps between binding sites within the tunnel network, rather than being solely dependent on a conjugated movement of carbon chains and bound ligands, as the single site alternating access model requires (Figs. 8a, 9; Tables 1, 2). Transient communication between the tunnels and the intermediate cavities will facilitate ligand flow through the transporter by a combination of diffusion within the tunnels and tunnel wall coalescence with neighbouring cavities. This latter activity opens gates between neighbouring regions of the pathway. Even if the channel never becomes wholly patent, ligands may still traverse the transporter in stages. Such stepped ligand interchanges are most likely to be driven as a result of thermally generated reptation of the carbon chains or amino acid side chains, (Langosch and Arkin 2009) which may be generated by interactions with the lipid bilayer in which the transporter is suspended. The ligands are as free to move between sites within the transporter tunnels (Figs. 9, 10). As the ligand affinity is, according to our findings with Autodock Vina, higher in the protein core than the periphery (Fig. 1), the longer residence time in the central region will increase the likelihood of transit when the narrow isthmus in the central region widens sufficiently to permit passage between the wide vestibular spaces on either side. This model is consistent with the transient transformation of transporters into channels, as observed in dopamine transporters (Kahlig et al. 2005) and with the recent model of transient opening of water channels in several classes of transporter (Li et al. 2013) and also with the possibility that intramolecular sequestration of ligands within transporters can generate water “cotransport” by osmosis (Naftalin 2008b; Zeuthen 2010). Similar properties have been shown to describe water movement within tunnels in small proteins, such as myoglobin. (Persson and Halle 2008 2013; Kaieda and Halle 2013). The transport phenomena described in these papers for water have several important similarities with sugar transport; namely very high temperature sensitivity and much higher exchange rates than net flux rates as previously discussed (Naftalin and De Felice 2012; Naftalin 2010).

### Simulation of Sugar Transport Via a Gated Channel Containing Sequential Binding Sites within Vestibules

**Fig. 11:**
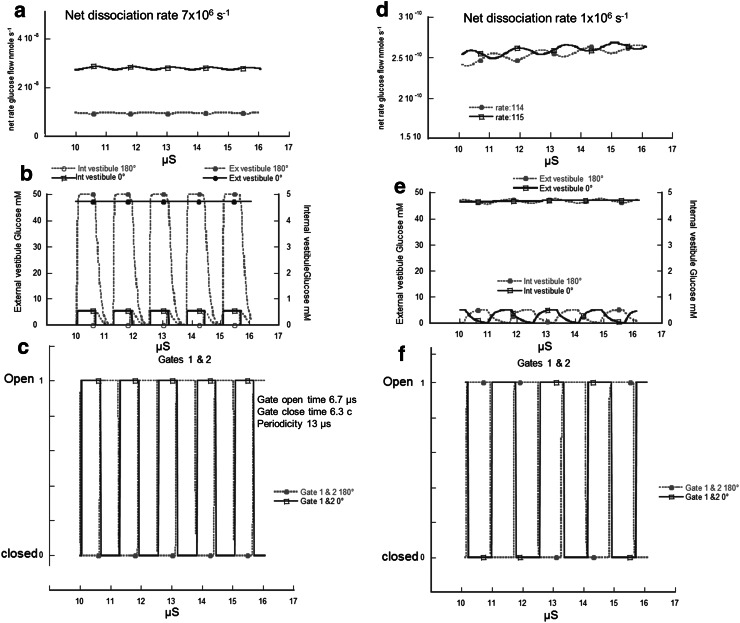
Comparison of simulated ligand fluxes and concentrations across the model transport system shown in Fig. 10 with high and low rates of ligand association/dissociation from the binding sites. a rates of net flow of ligand from outside to inside with 50 mM outside and zero inside with gates in phase (*blue*) and in antiphase (*red*). Gates opening in antiphase generate slower net transport than gates opening in phase. **b** and **c** ligand concentrations in external vestibule and internal vestibule in phase (*blue*) and during antiphase gate opening and closure. Note that when gate 1 closes ligand concentrations fall rapidly when gate 1 closes and gate 2 opens in antiphase (*red*). **d** Ligand net flux similar in phase and antiphase when ligand association and dissociation rates are comparable with gate opening rates in contrast with when association/dissociation rates are faster (*a*). **e** and **f** slow dissociation rates leave residual ligand within both vestibules during open periods in both in phase and antiphase gate sequences (Color figure online)

As explained in Methods, altering the ligand association rates *k*_1_, whilst holding *K*_D_s constant allows the frequency of ligand association and dissociation rates to be altered independently of gating rates. Assuming a gating frequency of approximately 10^6^ Hz for both gates, (Fig. 11c, f) the effects of changing the pseudo-first-order association rate, *k*_1_ from 10^8^ s^−1^ to 10^6^ s^−1^ causes a qualitative effect on the net transport rate as well as reducing net transport by approximately 100-fold (Fig. 11a, d). When the ligand association rates are faster than gating frequency, then altering the gate chronicity from in phase to antiphase reduces net glucose flux across the network by 65 %, (Fig. 11b); whereas if the ligand association rates are comparable, or less than the gating frequency, then phase alteration of the gates has a negligible effect on net flux (Fig. 11e). The reason for this is that slow ligand clearance from the vestibules and sites masks the oscillations in ligand concentrations within the vestibules due to gating, as is illustrated by the red (antiphase) and blue (in phase) lines in (Fig. 11b, e) showing the large changes in external and internal vestibular sugar concentrations with high rates of ligand association dissociation (Fig. 11b), in comparison with gating rates and slower rates (Fig. 11d). The model illustrates the crucial point that gating at high rates will not alter rates of ligand flow across a multisite network (Fig. 11d). During the interim period since first submitting this paper (Stelzl et al. 2014) have published a paper on LacY, another MFS transporter, which hints that the Jardetzky model may not be entirely appropriate as a description of how this cotransporter operates. Instead they suggest an “airlock” model in which two gates at either end of the central channel open sequentially. This is very similar to the model we have proposed.

## Conclusions

Multiple xylose docking sites are demonstrated on all the available conformers of XylE crystals. The sites lie within intramolecular tunnels that vary in length and volume between conformers. Conformer superposition demonstrates that the tunnels and cavities coalesce to form a transient intramolecular channel spanning the entire length of the transporter. These small-scale changes in position within the transmembrane helices can lead to staged diffusive transport between adjacent sites within these channels as tunnels and cavities coalesce. The presence of destabilizing amino acid sequences close to the central binding site may promote transport by undulations of the narrow sections of the channel.

## Electronic supplementary material

Below is the link to the electronic supplementary material.

Supplementary material 1 (DOCX 11 kb)

Supplementary material 2 (GIF 2192 kb)

Supplementary material 3 (GIF 973 kb)

Supplementary material 4 (GIF 1107 kb)
