# Supplementary material for: Reptation-Induced Coalescence of Tunnels and Cavities in Escherichia Coli XylE Transporter Conformers Accounts for Facilitated Diffusion
Source: J Membr Biol. 2014 Aug 28;247(11):1161–79. doi: 10.1007/s00232-014-9711-7 (PMC4207944; doi:10.1007/s00232-014-9711-7)
Supplement: Supplementary file 1 — Supplementary material 1 (DOCX 11 kb) [file 232_2014_9711_MOESM1_ESM.docx]

**Supplement Figure 1** **Views of the large tunnels mapped as a mesh of varying colours using SwissProt Deepview’s roller ball method of all eight of the available conformers of XylE.** The estimated total volumes of the tunnels at the internal surface and external tunnels in nm^3^ are shown beside each panel. The superimposed tunnel maps are shown in the bottom right panel. The nested tunnels generate a fully spanning channel through the transporter.

**Supplement Figure 2.** Unstructured regions of Xyl E conformers showing color coding as in Figure 1.

**Supplement Figure 3** Tunnel 1 extends from mauve and blue speckled filled tunnel starting in the depths of the external vestibule to the internal I vestibule (blue). The longer branch starts from the same point as tunnel 1 diverges in the central zone and doubles to the external vestibule as traced by the mauve line. The tunnel radii are mapped as functions of the tunnel lengths outputs are obtained directly from MOLE2. The probe radius used with the tunnel finding MOLE 2 for the tunnels illustrated in Figure 8c was 3.5Å. The bottleneck in tunnel 1 in the upper straight tunnel delineated by the blue line at a distance of 8-9 Å from the tunnel opening, close to F290,V422, W 448,M451 has a radius of 1.2 Å. The second bottleneck with a radius of 1.25 Å is close to L172 and L326 near the Y junction in tunnel 2 and the third also in tunnel 2 has a similar small radius close to W445 and M356. Altered rotamer positions can easily of all the amino acids with bulky side chains can readily affect the pore radii. In Figure 6a and 6c it is demonstrated that these choke point positions coincide with regions of high mobility.
