# Supplementary figures and images for: Reptation-Induced Coalescence of Tunnels and Cavities in Escherichia Coli XylE Transporter Conformers Accounts for Facilitated Diffusion
Source: J Membr Biol. 2014 Aug 28;247(11):1161–79. doi: 10.1007/s00232-014-9711-7 (PMC4207944; doi:10.1007/s00232-014-9711-7)

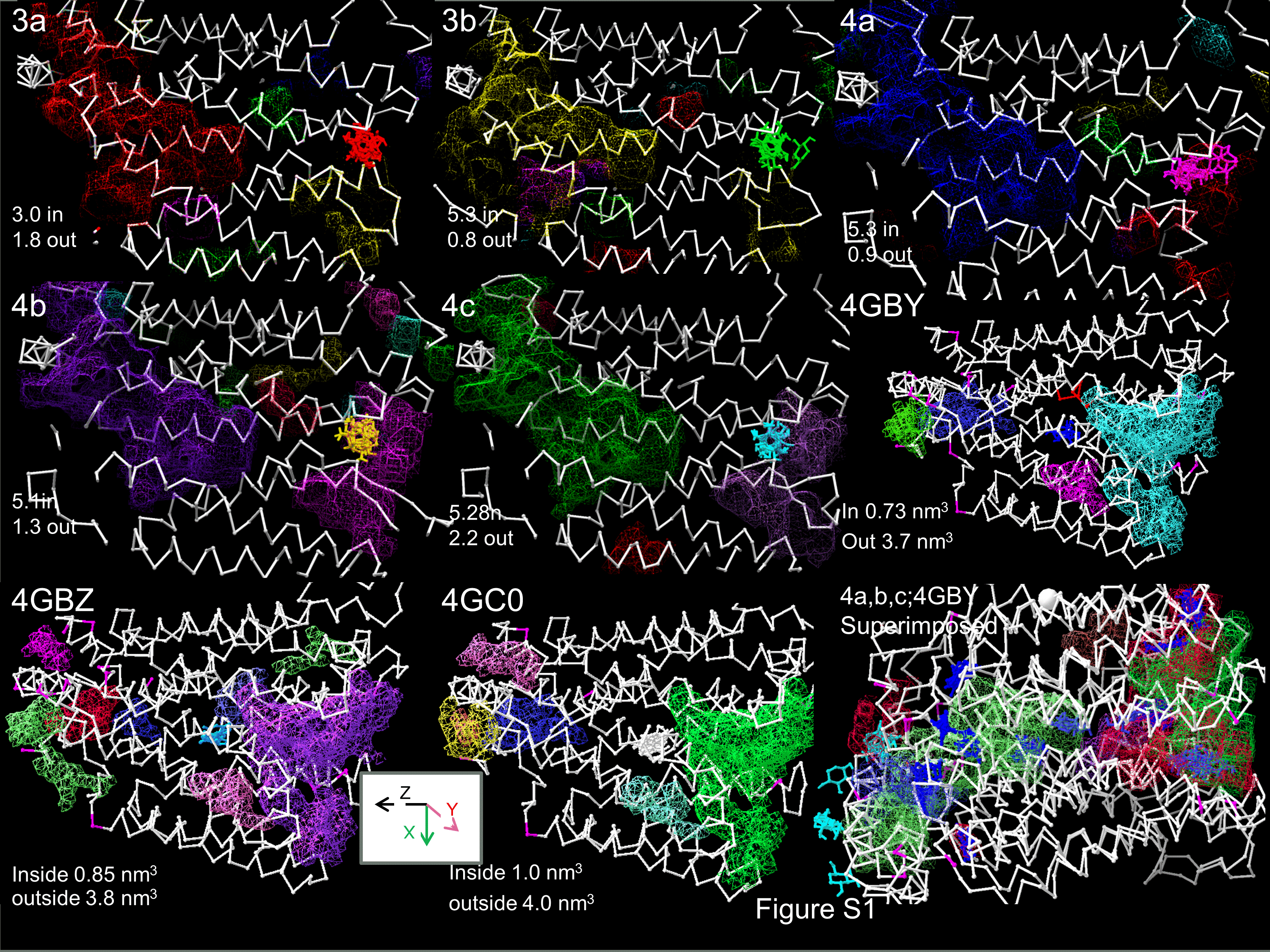

Supplement: Supplementary file 2 — Supplementary material 2 (GIF 2192 kb) [file 232_2014_9711_MOESM2_ESM.gif]

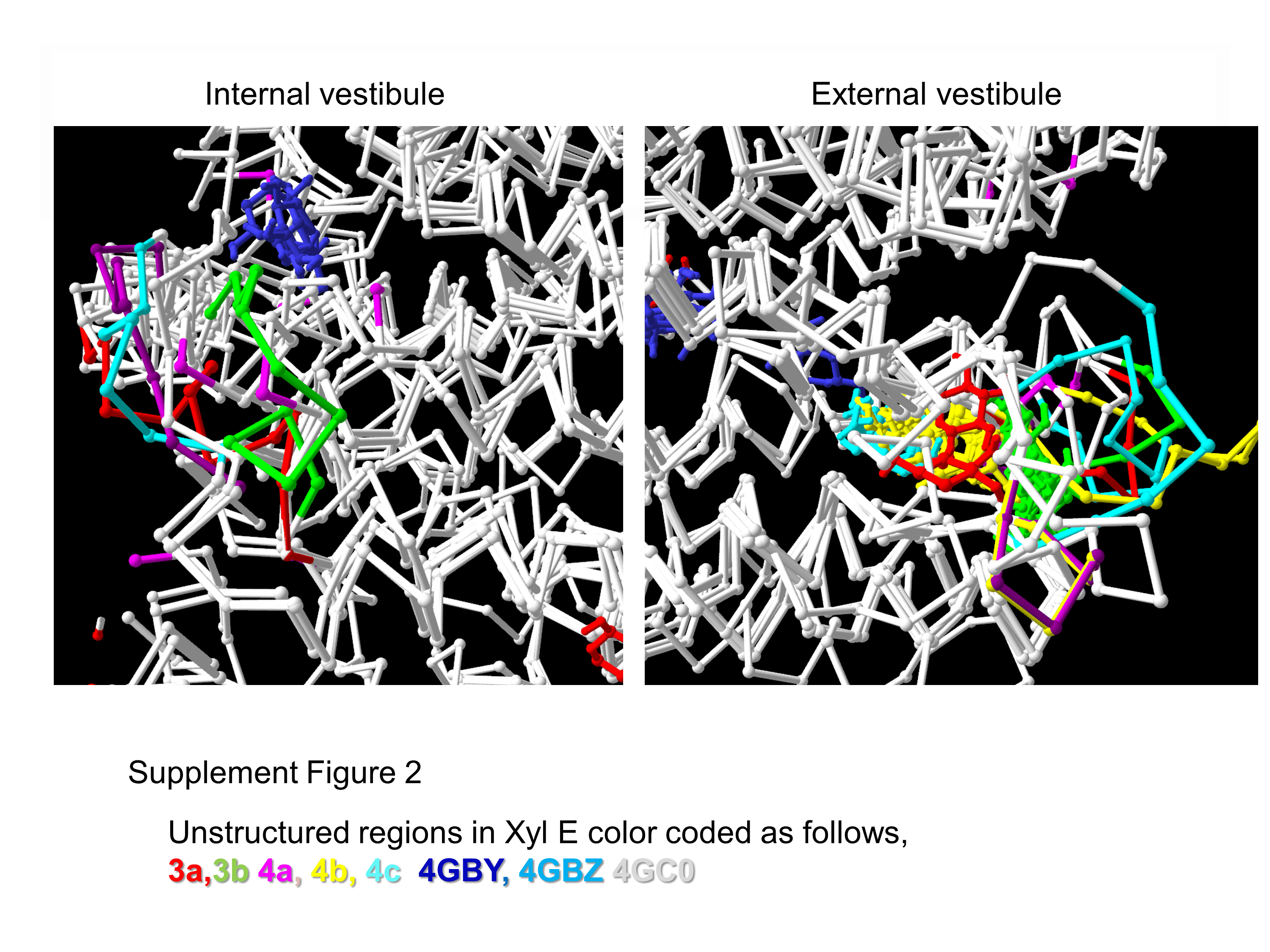

Supplement: Supplementary file 3 — Supplementary material 3 (GIF 973 kb) [file 232_2014_9711_MOESM3_ESM.gif]

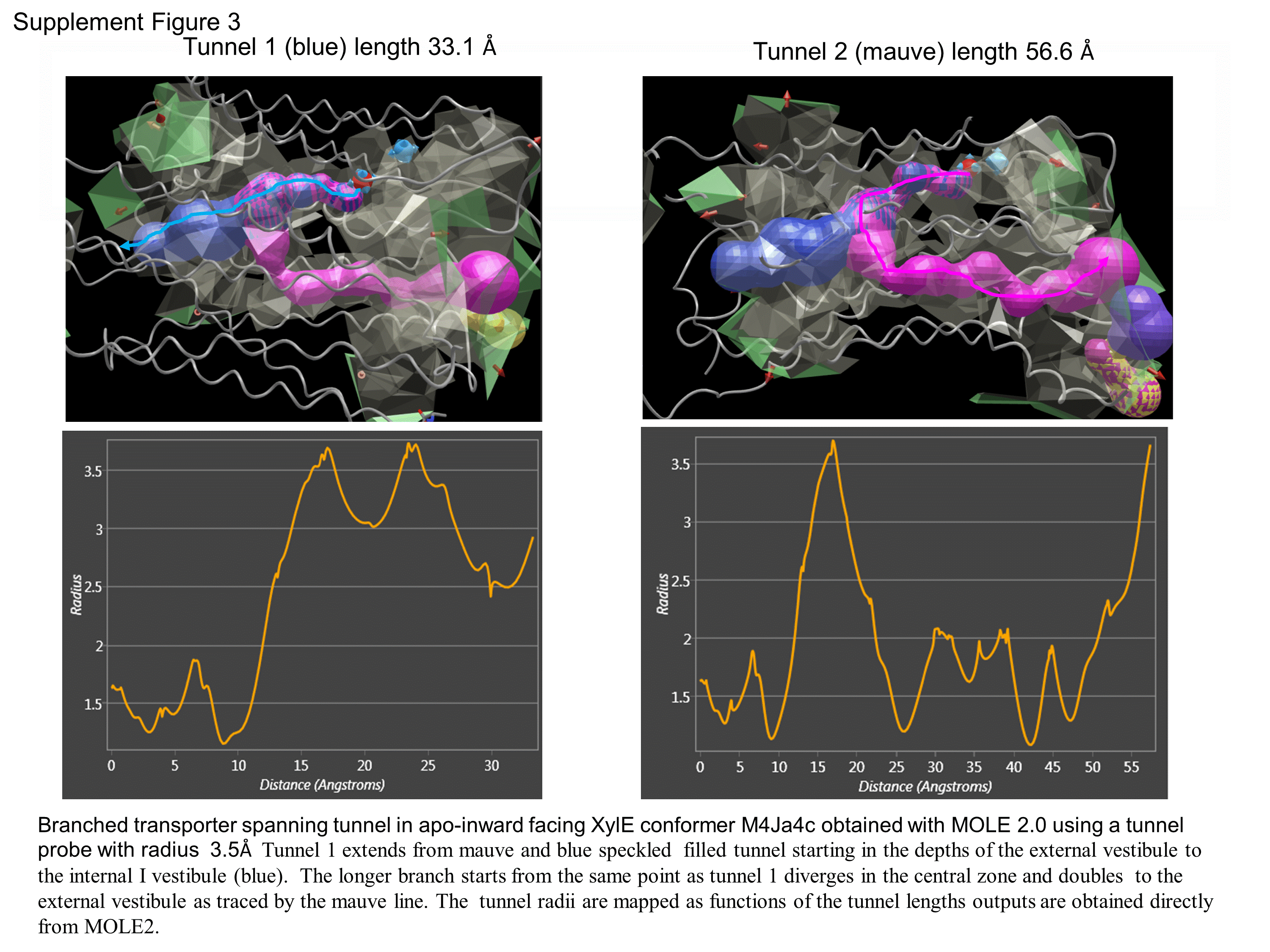

Supplement: Supplementary file 4 — Supplementary material 4 (GIF 1107 kb) [file 232_2014_9711_MOESM4_ESM.gif]
